# Contribution of syndecans to cellular uptake and fibrillation of α-synuclein and tau

**DOI:** 10.1038/s41598-019-53038-z

**Published:** 2019-11-12

**Authors:** Anett Hudák, Erzsébet Kusz, Ildikó Domonkos, Katalin Jósvay, Alpha Tom Kodamullil, László Szilák, Martin Hofmann-Apitius, Tamás Letoha

**Affiliations:** 1 Pharmacoidea Ltd., Szeged, H-6726 Hungary; 2 Biological Research Centre of the Hungarian Academy of Sciences, Szeged, H-6726 Hungary; 3 Fraunhofer Institute for Algorithms and Scientific Computing (SCAI), Sankt Augustin, 53754 Germany; 4 Szilak Laboratories, Bioinformatics and Molecule-Design, Szeged, H-6723 Hungary

**Keywords:** Glycobiology, Protein aggregation, Protein translocation

## Abstract

Scientific evidence suggests that α-synuclein and tau have prion-like properties and that prion-like spreading and seeding of misfolded protein aggregates constitutes a central mechanism for neurodegeneration. Heparan sulfate proteoglycans (HSPGs) in the plasma membrane support this process by attaching misfolded protein fibrils. Despite of intense studies, contribution of specific HSPGs to seeding and spreading of α-synuclein and tau has not been explored yet. Here we report that members of the syndecan family of HSPGs mediate cellular uptake of α-synuclein and tau fibrils via a lipid-raft dependent and clathrin-independent endocytic route. Among syndecans, the neuron predominant syndecan-3 exhibits the highest affinity for both α-synuclein and tau. Syndecan-mediated internalization of α-synuclein and tau depends heavily on conformation as uptake via syndecans start to dominate once fibrils are formed. Overexpression of syndecans, on the other hand, reduces cellular uptake of monomeric α-synuclein and tau, yet exerts a fibril forming effect on both proteins. Data obtained from syndecan overexpressing cellular models presents syndecans, especially the neuron predominant syndecan-3, as important mediators of seeding and spreading of α-synuclein and tau and reveal how syndecans contribute to fundamental molecular events of α-synuclein and tau pathology.

## Introduction

Misfolded proteins are key culprits in the pathomechanism of neurodegenerative disorders such as Alzheimer’s (AD) and Parkinson’s disease (PD)^1–3^. Currently untreatable beyond late stage symptomatic therapy, these invariably progressive disorders are increasing in prevalence (presently 45 million people worldwide, expected to reach 76 million in 2030) and have enormous impact on healthcare systems^4–6^. Disease modifying therapies slowing or halting disease progression in AD and PD are therefore major unmet medical needs^7,8^.

Recent evidence suggests that α-synuclein (α-syn) and tau fibrils have the capacity to spread from one cell to another and thereby induce neurodegeneration^9–11^. A thorough understanding of molecular and cellular mechanisms underlying propagation and prion-like spreading of α-syn and tau fibrils is likely to be a rich source of innovative targets for the development of novel disease modifying therapies for both AD and PD^12^.

Growing scientific data suggest the major role of cell surface heparan sulfate proteoglycans (HSPGs) in cellular attachment and subsequent internalization of α-syn and tau fibrils^13–16^. These findings highlight the potential effects of HSPGs on cellular pathophysiology driving neurodegeneration. Due to their highly versatile and sulfated glycosaminoglycan (GAG) chains, cell surface HSPGs interact with a plethora of ligands, including growth factors, cytokines along with several bacteria and viruses, thus modulating cellular metabolism, transport and signal transduction^17–20^. As α-syn and tau have been shown to interact with HSPGs, exploration of these interactions might help scientists to identify viable targets for future therapeutic interventions against neurodegeneration^13,14^. These interactions involve proteoglycans’ HS chains defined by their sulfation patterns^20^. The sulfation pattern of HS contributes significantly to the polysaccharide structural diversity and is critically involved in the binding of α-syn and tau, along with other ligands^17,21–26^. Although most cells express more than one HSPG at their cell surface, several lines of evidence indicate that HS chains attached to different core proteins on the same cell surface have the same sulfation patterns^27–31^. Investigating the interaction of various HSPG representatives with α-syn and tau in a given cell type would thus reveal further details of these interactions beyond sulfation specificity.

Analysis of postmortem human brain tissues from AD patients showed significant increase in the amounts of several HSPGs, including syndecan-3 and -4, two members of the syndecan (SDC) family of transmembrane HSPGs^32^. Clinical studies also highlight the correlation between neurodegeneration patterns and SDC gene expressions^33^. Studying the fibrillation and intracellular uptake of amyloid-β(1–42), we found that the overexpression of SDCs, especially the neuron predominant SDC3, markedly supports amyloid-β pathology by triggering fibrillation and intracellular delivery of fibrillar amyloid-β(1–42)^17^.

The four member family of SDCs are the only transmembrane HSPGs^17,34^. SDCs show tissue specific expression: SDC1 is expressed by epithelia and plasma cells, SDC2 by cells of mesenchymal origins (endothelials and fibroblasts), while SDC3 is enriched in neurons. SDC4, on the other hand, is more ubiquitous^34–36^. SDC core proteins are made of a conserved short, one span transmembrane domain and the approximately 30 amino acid length cytoplasmic domain^37^. Through their cytoplasmic domains, SDCs influence a large number of signaling cascades, regulated by the transmembrane domain-induced oligomerization, a unique regulatory mechanism in SDC signaling^17,36,38–42^. It is worth noting, that difference in oligomerization tendency seems to influence the function of each SDC^39,41^. The N-terminal, divergent extracellular domains (ectodomains) contain three GAG attachment sites for HS near the N terminus and may bear chondroitin sulfate (CS) at their juxtamembrane region^17,36,43^. Specific contacts between sulfated regions of the GAG chains and basic residues of proteins enable SDCs to interact with myriad of extracellular ligands and transmit signals from the extracellular space towards the cellular interior^44–46^. SDC binding also promotes oligomerization of bound ligands, resulting from the multivalent nature of the ligand-binding sites on SDCs (i.e. the multiple GAG chains, each with multiple ligand-binding sites) and from core-protein-mediated oligomerization of the SDCs themselves^40,47^. One of the exciting feature of SDCs’ GAG chains is that the HS fine structure reflects the cellular source of the SDC rather than SDC type: therefore the same SDC isoform can have distinct ligand binding properties in different cell lines^26,31^. Besides its GAG attachment sites, SDC3 also possesses a number of potential sites for O-linked glycosylation (resembling a mucin-rich domain), while the SDC4 ectodomain contains a cell-binding domain (CBD) mediating cell to cell attachment^48–51^.

Membrane SDCs also act as endocytic receptors, and undergo constitutive as well as ligand-induced endocytosis^17,36,52,53^. SDC-mediated endocytosis appears to occur independently of clathrin and caveolin, but in a lipid raft-dependent manner: ligands or specific antibodies induce clustering and redistribution of SDCs to lipid rafts, thus stimulating a lipid raft-dependent, but clathrin- and caveolae-independent endocytosis of the SDC core protein^17,36,54–56^. The lipid raft dependence of α-syn and tau spreading has been already proposed^57,58^, while accumulation of flotillin 1 - the marker of lipid rafts - in tangle-bearing neurons of AD has also been reported^17,59^.

Many pathogens and proteins exploit SDC-mediated endocytosis to translocate into cells^60–62^. Moreover a number of macromolecular drug delivery agents, including cell-penetrating-peptides (CPPs) and lipoplexes also utilize membrane SDCs to enter cells and transport attached cargoes intracellularly^17,63–67^. The observation that macromolecules internalized via SDC-mediated uptake can dodge complete degradation in the lysosomes and exert their bioactivity intracellularly demonstrates why SDC-mediated uptake of misfolded protein aggregates could be detrimental in neurodegeneration^17,36,63,64,68^.

Over the years, our research group has been exploring the protein delivery potential of SDCs^17,36,63,64,68^. Several of the peptidic drug delivery agents, including the HIV-1 derived Tat peptide, enter the cells via HSPG-mediated macropinocytosis and show cellular entry characteristics similar to α-syn and tau, further supporting the relevance of SDCs in α-syn and tau internalization^12,36,47,69^.

Considering the evidence of SDCs’ involvement in neurodegeneration, we carried out extensive studies to explore the role and contribution of SDCs to fibrillation and cellular internalization of α-syn and tau. To minimize the interference with other HSPGs, we applied SDC overexpressing cellular models created in K562 cells, a cell line with reportedly low amount of endogenous HS (limited to betaglycan and a small amount of SDC3), hence enabling the exact assessment of SDCs’ contribution to cellular uptake and aggregation of α-syn and tau^17,70–73^. As K562 cells express no caveolin-1, the major component of caveolae, K562 cells bear limited capacity for caveolae formation, thus caveolae-mediated endocytosis^17,74,75^. Application of stable SDC transfectants created in K562 cells therefore helped to study the specific effect of SDC overexpression on α-syn and tau uptake, while avoiding the interference with other HSPGs or caveolar endocytosis^17^. Quantitative flow cytometric analyses also assisted the accurate measurement of SDCs’ involvement in α-syn and tau uptake, while assays with structural mutants exposed the role of SDC domains in α-syn or tau fibril uptake^17^. Thioflavin T (ThT) fluorescence assays and scanning electron microscopy served to explore the effect of SDCs on α-syn and tau fibrillation, a molecular event necessary for the prion-like spreading of the proteins. Observations acquired on stable SDC transfectants (created in the K562 cell line) were also supported by studies conducted on differentiated and undifferentiated SH-SY5Y cells.

The gathered cellular data elucidate SDCs’ contribution to the seeding and spreading of α-syn and tau and show how overexpression of SDCs, irrespective of their cellular source, can trigger fundamental molecular events in α-syn and tau pathology.

## Results

### Contribution of SDCs to α−syn and tau uptake and fibrillation

**Figure 1:**
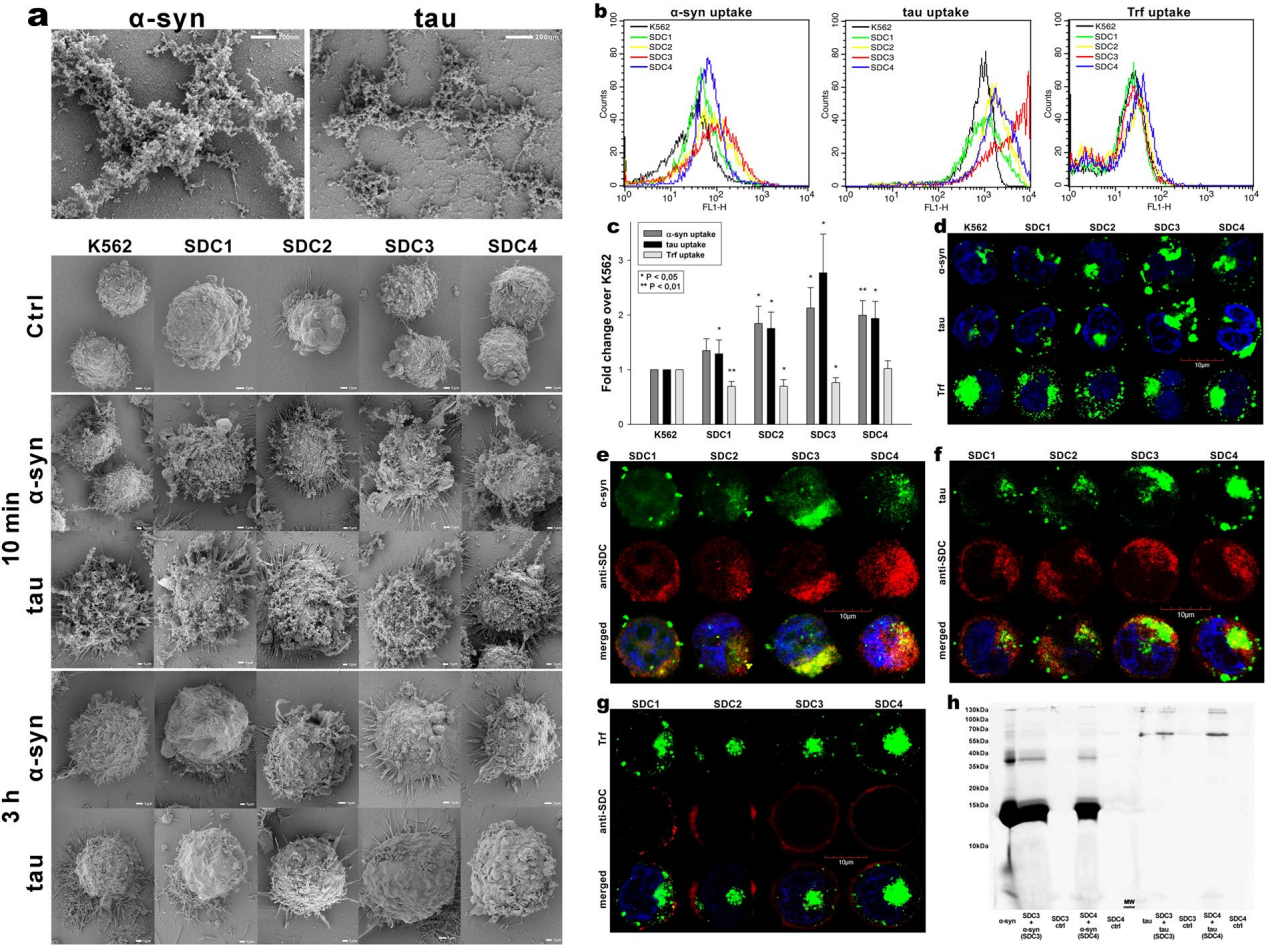
Cellular uptake of α−syn and tau fibrils into SDC transfectants. WT K562 cells and SDC transfectants were incubated with either of the FITC-labeled fibrils (α−syn or tau at a concentration of 5 µM monomer equivalent) or Trf (25 µg/ml) for 3 h at 37 °C. Cellular uptake of the fibrils and Trf was then analyzed with flow cytometry and microscopy. **(a)** Scanning electron microscope visualization of α−syn and tau fibrils, along with WT K562 cells and SDC transfectants treated with the fibrils at 10 min and 3 h of incubation. (**b**) Flow cytometry histograms showing intracellular fluorescence of WT K562 cells and SDC transfectants, following 3 h incubation with fluorescent α−syn or tau fibrils or Trf. **(c)** Detected fluorescence intensities were normalized to fibril (α−syn or tau) or Trf-treated WT K562 cells as standards. The bars represent mean ± SEM of five independent experiments. Statistical significance vs standards was assessed by analysis of variance (ANOVA). *p < 0.05 vs standards; **p < 0.01 vs standards. **(d)** CLSM visualization of fibril (α−syn, tau) and Trf uptake. (**e**–**g**) Colocalization of α−syn or tau fibrils and SDCs. SDC transfectants treated with either of the fluorescent fibrils (α−syn or tau) or Trf for 3 h were permeabilized and treated with the respective APC-labeled SDC antibody. Nuclei of cells were stained with DAPI and cellular uptake was then analyzed with CLSM. Representative images of three independent experiments are shown. Scale bar = 10 μm. **(h)** SDS-PAGE showing FITC-labeled α-syn and tau fibrils immunoprecipitated with either SDC3 or SDC4 antibodies from extracts of SDC3 and SDC4 transfectants. Lane 1: 0.5 ug of FITC- α-syn; Lane 2: immunoprecipitate of FITC-α-syn-treated stable SDC3 transfectants; Lane 3: immunoprecipitate of untreated SDC3 transfectants; Lane 4: immunoprecipitate of FITC-α-syn-treated SDC4 transfectants; Lane 5: immunoprecipitate of untreated SDC4 transfectants; Lane 6: Molecular weight (MW) marker; Lane 7: 0.5 µg of FITC-tau; Lane 8: immunoprecipitate of FITC-tau-treated SDC3 transfectants; Lane 9: immunoprecipitate of untreated SDC3 transfectants. Lane 10: immunoprecipitate of FITC-tau-treated SDC4 transfectants; Lane 11: immunoprecipitate of untreated SDC4 transfectants. Standard protein size markers are indicated on the left.

Although HSPGs have been already acknowledged as major players in the cellular uptake of α-syn and tau fibrils, evidence on the contribution of specific SDC isoforms to cellular internalization of these misfolded proteins is still waiting to be explored^76^. In order to measure SDCs’ contribution to aggregation and cellular spreading of α-syn and tau, with minimal interference with other HSPGs or caveolae-mediated endocytosis, stable SDC transfectants were created in K562 cells, a cell line with reportedly low HSPG expression, along with no detectable levels of caveolin-1, the main component of caveolae^17,70–73,77^. The low membrane HSPG expression of K562 cells and their inability to form caveolae – required for caveolar endocytosis - makes K562 an ideal human model cell line to study the effects of SDC overexpression without the interference of other HSPGs or caveolae-mediated endocytosis^17^. Since cell surface HS has been already recognized as a major contributor to cellular binding and uptake of protein aggregates, SDC transfectants were standardized according to their HS expression (as noted in our previous study, contrary to HS, we could not detect any CS on wild-type [WT] K562 cells or any of the SDC transfectants)^17^. Thus SDC transfectants with membrane HS levels alike were selected and together with WT K562 cells, incubated with FITC-labeled α-syn or tau fibrils for 3 h. The state of fibrils added, along with the surface of fibril-treated cells was also examined with electron microscopy (Fig. 1a). Thus scanning electron microscopy revealed that the high number of fibrils on the cell surface of fibril-treated cells at the start (i.e. 10 min) of the incubation period was reduced at 3 h, suggesting the onset of intense internalization. (Previous reports on the very moderate *in vitro* degradation of α-syn or tau fibrils in the extracellular space also suggest that internalization is the major cellular process responsible for the clearance of extracellular α-syn or tau aggregates^78–81^). Thus the intracellular fate of the fibrils was then studied with quantitative flow cytofluorometric and microscopic assays^17^. Flow cytometric measurement of uptake was conducted by adding trypan blue (dissolved at a concentration of 0.25% in ice-cold 0.1 M citrate buffer) 1 min before the analyses, thus extracellular fluorescence of surface bound fluorescent proteins was quenched, hence enabling the exact assessment of the internalized proteins^17,36,82^. The rate of classical endocytic pathways was simultaneously detected by measuring the uptake of fluorescently labeled transferrin (Trf), the marker of clathrin-mediated endocytosis^83^. As Fig. 1b,c show, SDCs - especially the neuron predominant SDC3 - increased the cellular uptake of fibrils, while internalization of Trf, the marker of clathrin-mediated endocytosis was reduced by SDC1-3 (and left unaffected by SDC4) suggesting that SDC mediated uptake of the fibrils occur through clathrin-independent routes. Microscopic studies revealed similar pattern as flow cytometry: namely that compared to WT K562 cells, fibril-treated SDC transfectants exhibited higher intracellular fluorescent signals (Fig. 1d). CLSM (confocal laser scanning microscopy) colocalization studies then revealed apparent intracellular colocalization of SDCs with α-syn and tau fibrils (the Mander’s overlap coefficients [MOC] for SDCs with α-syn and tau exceeded 0.7, an indicator of significant colocalization [Supplementary Fig. S1]), suggesting the common intracellular pathway SDCs and α-syn or tau fibrils follow during internalization (Fig. 1e,f). Unlike α-syn or tau, Trf - demonstrating the characteristic features clathrin-mediated endocytosis (i.e. vesicle-like intracytoplasmic structures) - exhibited very weak colocalization with any of the SDCs after 3 h of incubation (i.e. MOCs < 0.4 for all SDCs), indicating that SDC-mediated pathways occur independent of clathrin (Fig. 1g and Supplementary Fig. S1). Co-immunoprecipitation of fibril treated SDC transfectants also confirmed that α-syn or tau fibrils bind SDC3 and SDC4 (Fig. 1h).

**Figure 2:**
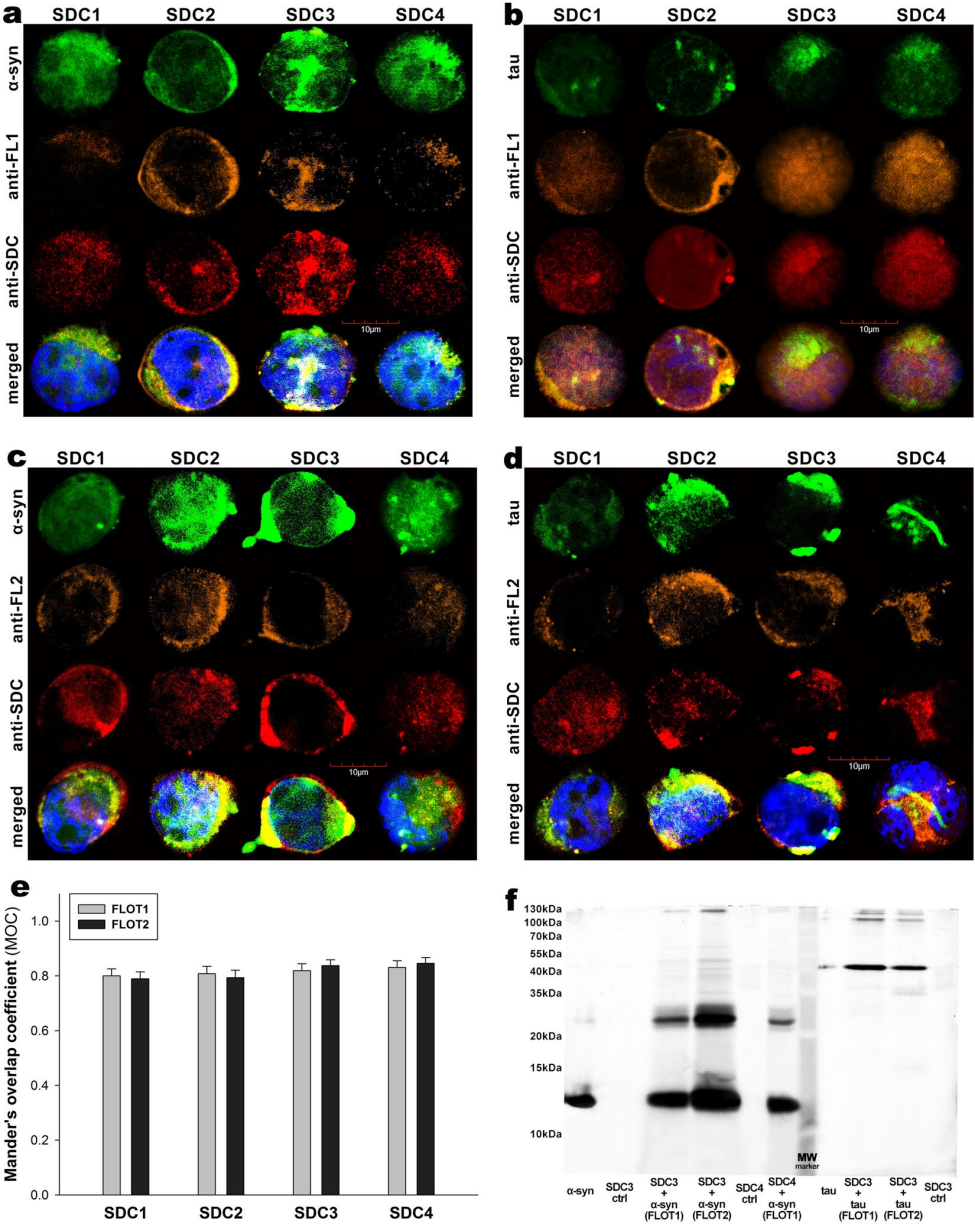
Colocalization of α−syn and tau fibrils with SDCs and flotillins. SDC transfectants, treated with fluorescently labeled α−syn or tau fibrils (at a concentration of 5 µM monomer equivalent) for 3 h at 37 °C, were permeabilized and treated with the respective APC-labeled SDC antibodies, along with either flotillin 1 (FLOT1) or 2 (FLOT2) antibodies (both Alexa Fluor 546-labeled). Nuclei of cells were stained with DAPI and colocalization was then analyzed with CLSM. **(a**–**d)** CLSM images of SDC transfectants treated with either of the fluorescent fibrils (α−syn or tau), one of the flotillin antibodies (FLOT1 or 2) and a respective APC-labeled SDC antibody. Representative images of three independent experiments are shown **(e)** MOC ± SEM for the overlap of SDCs with either FLOT1 or FLOT2 was calculated by analyzing 21 cellular images (7 images per sample, experiments performed in triplicate). **(f)** SDS-PAGE showing fluorescent α−syn or tau immunoprecipitated with either of the flotillin antibodies from extracts of stable SDC3 and SDC4 transfectants. Fluorescent α−syn and tau were detected by Uvitec’s Alliance Q9 Advanced imaging platform. Lane 1: 0.5 ug of FITC-α−syn; Lane 2: immunoprecipitate of untreated SDC3 transfectants (controls); Lane 3–4: immunoprecipitate of FITC-α−syn-treated, stable SDC3 transfectants; Lane 5: immunoprecipitate of untreated SDC4 transfectants (controls) Lane 6: immunoprecipitate of FITC-α−syn-treated, stable SDC4 transfectants; Lane 7: Molecular weight (MW) marker; Lane 8: 0.5 ug of FITC-tau; Lane 9–10: immunoprecipitates of FITC-tau-treated stable SDC3 transfectants. Lane 11: immunoprecipitate of untreated SDC3 transfectants (controls). Standard protein size markers are indicated on the left.

HSPGs are internalized via a lipid raft-dependent, yet clathrin- and caveolin-independent pathway^17,52,84^. The flotillin family of membrane proteins (i.e. flotillin 1 [FLOT1] and flotillin 2 [FLOT2]) have been acknowledged as markers of lipid rafts^85^. The involvement of lipid rafts in the SDC-mediated internalization of α-syn and tau fibrils was justified with both microscopic and Co-IP studies (Fig. 2a–f): CLSM studies demonstrated apparent intracellular colocalization of both flotillins with SDCs, along with α-syn and tau fibril in SDC transfectants (MOCs of FLOT1 and 2 with SDCs ~0.8), while Co-IP studies confirmed the colocalization of SDC3 and 4 with flotillins and α-syn and tau fibrils (Fig. 2a–f). It is worth noting that in our recent affinity-based proteomics study, FLOT1 and -2 were detected in a pull-down experiment of SDC4, further supporting the findings of the CLSM colocalization studies^17^.

### Effect of SDC domains on cellular internalization of α-syn and tau fibrils

**Figure 3:**
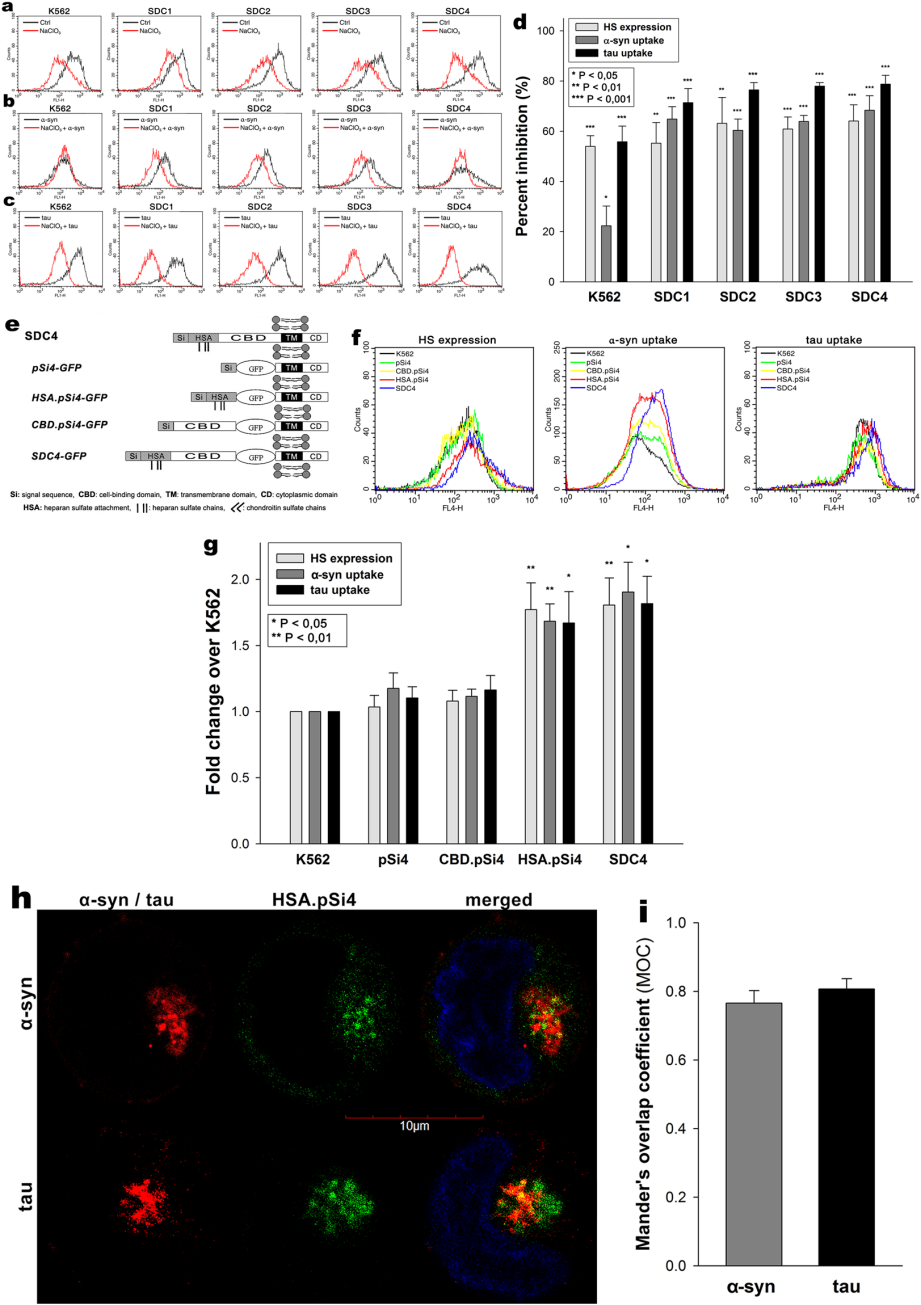
Effect of SDC domains on α−syn and tau fibril uptake. (**a**) Flow cytometry histograms representing HS expression of WT K562 cells or SDC transfectants after NaClO_3_ treatment. **(b**,**c)** Flow cytometry histograms representing intracellular fluorescence of fluorescent (FITC) fibril (α−syn or tau) treated WT K562 cells and SDC transfectants preincubated with or without NaClO_3_. **(d)** The effect of NaClO_3_ were expressed as percent inhibition, calculated with the following formula: [(X − Y)/X] × 100, where X is the fluorescence intensity obtained on cells treated with either of the fibrils in the absence of NaClO_3_ and Y is the fluorescence intensity obtained on cells treated with either of the fibrils in the presence of NaClO_3_. The bars represent mean ± SEM of four independent experiments. Statistical significance vs controls untreated with NaClO_3_ was assessed by analysis of variance (ANOVA). *p < 0.05 vs standards; **p < 0.01 vs standards; ***p < 0.001 vs standards. **(e**–**i)** Contribution of various parts of the SDC4 ectodomain to fibril uptake. WT K562 cells and SDC4 mutants were treated with Alexa Fluor 633-labeled α−syn and tau fibrils at a concentration of 5 μM at 37 °C. **(e)** Schematic representation of SDC4 deletion mutants used in the study. **(f)** Flow cytometry histograms representing HS expression and intracellular fluorescence of WT K562 cells and SDC4 mutants treated with fluorescent α−syn and tau fibrils for 3 h. **(g)** Results of flow cytometric measurements. Detected fluorescence intensities were normalized to fibril-treated WT K562 cells as standards. The bars represent mean ± SEM of four independent experiments. Statistical significance vs fibril-treated WT K562 cells (standards) was assessed by analysis of variance (ANOVA). *p < 0.05 vs standards; **p < 0.01 vs standards. **(h)** CLSM visualization of HSA.pSi4 transfectants treated with fluorescent α−syn and tau fibrils for 3 h. Scale bar = 5 μm. **(i)** MOC ± SEM for the overlap of fluorescent α−syn and tau fibrils with HAS.pSi4 was calculated by analyzing 21 cellular images (7 images per sample, experiments performed in triplicate).

The involvement of SDC domains in the interaction with α-syn and tau fibrils were then explored in studies affecting various parts of the SDC ectodomain. Inducing undersulfation of SDCs’ HS chains with sodium chlorate (NaClO_3_), an inhibitor of protein sulfation^86^, significantly (p < 0.01) reduced on α-syn and tau fibril uptake in all cells lines, highlighting the importance of polyanionic HS chains in interaction with the proteins (Fig. 3a–d).

The HS chains of SDCs offer a binding site for a large number of macromolecular ligands. Besides HS chains, SDC4 also possess another binding site, the so-called cell-binding domain (CBD) mediating cell-to-cell attachment^17,36,68^. To study whether CBD influence the uptake of α-syn and tau fibrils, we created several structural mutants of SDC4, one of the isoform that induced significant increase in the uptake of α-syn and tau fibrils (Fig. 1b–d). Among the SDC4 structural mutants created (Fig. 3e), the CBD.pSi4 is made of the CBD and the secretion signal sequence (Si), but lacking any HS chains^17,36,68^. HSA.pSi4 mutants on the other hand lack the CBD, but contain the HS attachment site (HSA) with the HS chains, while pSi4 mutants possess a truncated extracellular domain made of only the Si of SDC4 (Fig. 3e)^17,36,68^. All of the above mentioned SDC4 mutants, along with one coding WT SDC4, were tagged with GFP at the juxtamembrane region and expressed in K562 cells^17^. Clones with equal extent of GFP, hence SDC expression were then selected with flow cytometry and – along with WT K562 cells as controls – treated with α-syn and tau fibrils for 3 h at 37 °C. Cellular uptake and attachment were then analyzed with flow cytometry, as well as confocal microscopy. Compared to WT K562 cells, WT SDC4 transfectants exhibited the most significant (p < 0.05) increase in α-syn and tau fibril uptake (Fig. 3f,g). Mutants without HS chains (i.e. pSi4 or CBD.pSi4) did not increas α-syn and tau uptake, showing the minor involvement of the signal sequence or the CBD domain in the interactions with α-syn or tau fibrils. On the other hand, mutants with a truncated ectodomain containing only the HSA with the HS chains increased fibril internalization at an extent similar to SDC4 transfectants, thus confirming the significance of polyanionic HS chains in the interactions with α-syn or tau fibrils. Microscopic studies also showed apparent intracellular colocalization of HSA.pSi4 with both α-syn and tau fibrils (MOC = 0.76 and 0.8, respectively), proposing that α-syn and tau fibrils are internalized bound to the HS chains of SDC4 (Fig. 3h,i).

**Figure 4:**
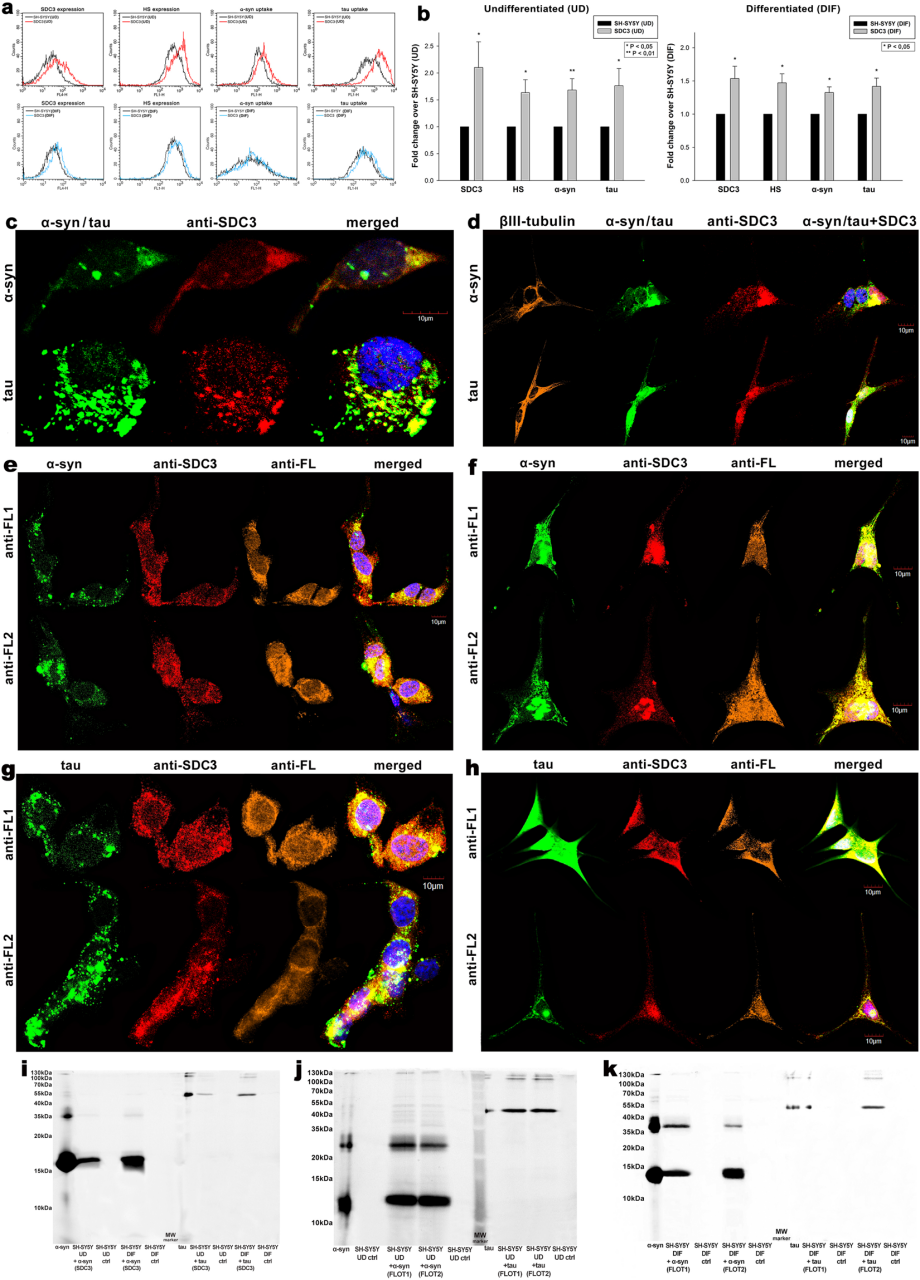
Effect of SDC3 overexpression on α−syn and tau fibril uptake in SH-SY5Y cells. SDC3 transfectants, created in either differentiated (DIF) or undifferentiated (UD) SH-SY5Y cells, were selected by measuring SDC3 expression with flow cytometry using APC-labeled anti-SDC3 antibody. HS expression of SDC3 transfectants, along with WT SH-SY5Y, was also measured with flow cytometry using anti-HS antibody. SDC3 transfectants and WT SH-SY5Y cells were treated with FITC-labeled α−syn and tau fibrils (at a concentration of 5 µM monomer equivalent) at 37 °C. Cells incubated with fluorescent α−syn and tau fibrils for 3 h were then processed for uptake studies. (**a**) Flow cytometry histograms representing SDC3, HS expression levels and intracellular fluorescence of WT SH-SY5Y cells and SDC3 transfectants treated with fluorescent α−syn or tau fibrils. (**b**) Fold change in SDC3 and HS expression, along with α−syn and tau uptake following SDC3 overexpression in undifferentiated (UD) or differentiated (DIF) SH-SY5Y cells. The bars represent mean ± SEM of six independent experiments. Statistical significance vs fibril (α−syn or tau) treated WT SH-SY5Y cells as standards was assessed by analysis of variance (ANOVA). *p < 0.05 vs fibril (α−syn or tau) treated WT SH-SY5Y cells as standards. **(c,d)** Colocalization of the fibrils with SDC3 in undifferentiated (**c**) or differentiated (**d**) SH-SY5Y cells. CLSM images of WT SH-SY5Y cells treated with either of the FITC-labeled fibrils (α−syn or tau), along with APC-labeled SDC3 antibody. In case of differentiated SH-SY5Y cells (DIF), neuronal differentiation was justified by staining the cells with neuron-specific human βIII-tubulin antibody and Alexa Fluor 546-labeled secondary antibody. Scale bar = 10 μm. **(e**–**h)** Colocalization of the fibrils with flotillins undifferentiated (**e,g**) or differentiated (**f**,**h**) SH-SY5Y cells. CLSM images of WT SH-SY5Y cells treated with either of the fluorescent fibrils (α−syn or tau), along with APC-labeled SDC3 antibody and either of the Alexa Fluor 546-labeled FLOT1 and FLOT2 antibodies. Scale bar = 10 μm. **(i**–**k)** SDS-PAGEs showing fluorescent α−syn or tau immunoprecipitated with either SDC3 or one of the flotillin antibodies from extracts of undifferentiated (UD) or differentiated (DIF) SH-SY5Y cells.

SDC3’s contribution to cellular entry of α-syn and tau fibrils were also analyzed in the more complex SH-SY5Y cell line. The effect of SDC3 overexpression - in both differentiated and undifferentiated SH-SY5Y cells – was thus studied on α-syn and tau fibril uptake. SDC3 overexpression – either in differentiated or undifferentiated SH-SY5Y cells - induced increased HS expression, along with increased internalization of both fibrils (Fig. 4a,b). Colocalization studies also confirmed the common intracellular pathway SDC3 and α-syn or tau fibrils follow during cellular internalization (Fig. 4c,d), with Mander’s overlap coefficients (MOCs) all (i.e. either between SDC3 and α-syn or SDC3 and tau) exceeding 0.7, indicating strong colocalization (Supplementary Fig. S2). The SDC3-mediated uptake of α-syn or tau fibrils in SH-SY5Y cells occur through lipid rafts, as shown by the apparent colocalization of the aggregates with flotillins (Fig. 4e–h with relevant MOCs shown in Supplementary Fig. S2b,c). Further Co-IP experiments also confirmed the colocalization of α-syn or tau fibrils with either SDC3 or flotillins, as both α-syn or tau could be immunoprecipitated with either SDC3 or flotillin 1 or 2 (Fig. 4i–k).

### Conformation dependence of SDC-mediated uptake

**Figure 5:**
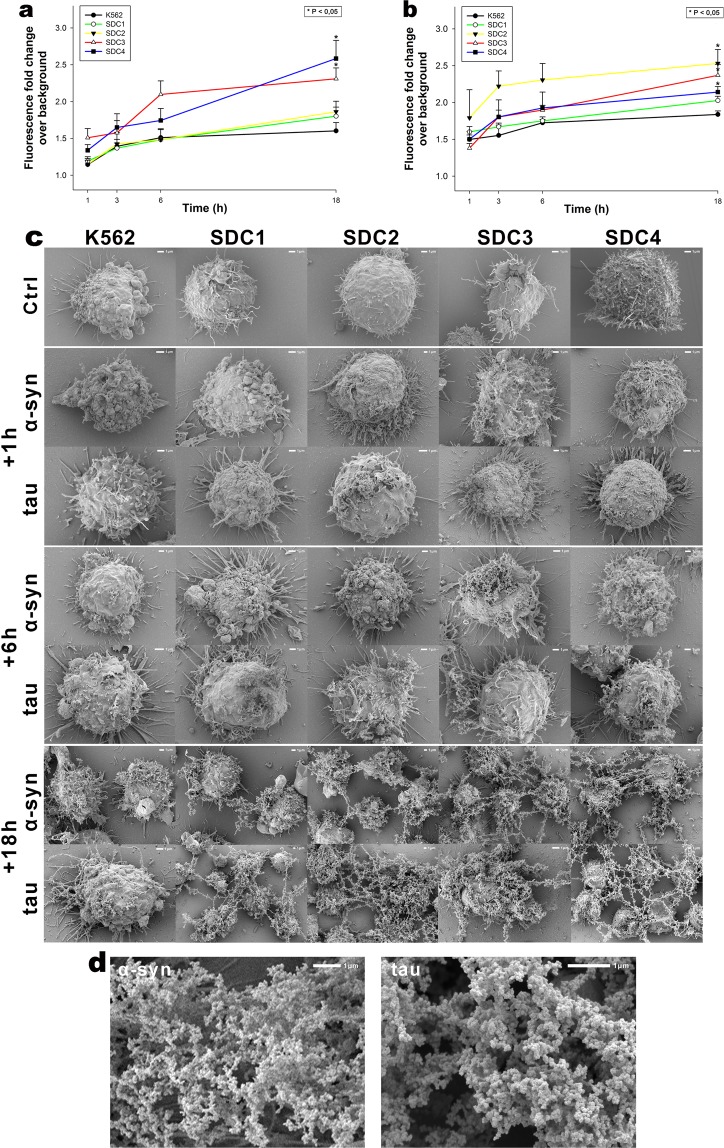
Effects of SDCs on α−syn and tau fibrillation. WT K562 cells and SDC transfectants were incubated with α−syn and tau monomers at a concentration of 5 μM for up to 18 h at 37 °C. (**a,b**) Kinetics of α−syn and tau fibrillation after 1 h **(a)** and 18 h **(b)**. 1, 3, 6 and 18 h after treatment with α−syn or tau monomers, the cells were treated with ThT at a concentration of 15 μM for 10 mins and fluorescence was measured. ThT fluorescence is expressed as fold change over background ThT fluorescence. The bars represent mean ± SEM of four independent experiments. Statistical significance vs α−syn or tau-treated WT K562 cells was assessed by analysis of variance (ANOVA). *p < 0.05 vs α−syn or tau treated WT K562 cells. **(c)** Scanning electron microscope visualization of cellular surface of WT K562 cells and SDC transfectants treated with α−syn or tau monomers for 1 h, 6 h, 18 h. Representative images of three independent experiments are shown. Scale bar = 1 μm. (**d**) High resolution electron microscopic visualization of fibrils formed after 18 h of incubation with SDC3 transfectants. Representative images of three independent experiments are shown. Scale bar = 1 μm.

Previous studies suggested that HSPG-mediated α-syn and tau uptake is heavily dependent on conformation, namely that fibrils enter the cells via HSPG-dependent pathways, while monomers and smaller oligomers enter the cells through other routes^87^. Thus we investigated SDCs’ effect on α-syn and tau fibrillation and uptake by incubating (freshly prepared) monomeric of α-syn and tau with SDC transfectants (established in K562 cells) and monitoring the fate of the monomers with fibrillation and uptake studies. ThT fluorescence studies quantitatively showed the fibrillation triggering effect of SDCs on α-syn and tau (Fig. 5). Thus SDC3 and to a lesser extent, SDC4 and triggered fibrillation of α-syn and tau over time (Fig. 5a,b). Simultaneous scanning electron microscopy studies also revealed the aggregation inducing effect of SDCs on surface attached α-syn or tau (Fig. 5c). Scanning electron microscopy showed the growing number of fibrillar α-syn or tau assemblies on the surface of SDC overexpressing cells. (Fig. 5c). The extent of fibril formation due to SDC overexpression, especially in the case of SDC2-4 is very spectacular: 18 h after addition of α-syn and tau monomers to SDC transfectants, scanning electron microscopy revealed widespread appearance of mature fibrils (i.e. larger than 2 µm) expanding transcellularly. Electron microscopic visualization of the structure of fibrils induced by SDC3, shown in Fig. 5d, reveals that in the very brief time of 18 h the overexpression of SDC3, along with SDC4 (and in case of tau, SDC2) triggered the formation of mature fibrils, thus highlighting the importance of fibrillation triggering effects of SDCs. It is also evident, that SDCs can induce the formation of neurodegeneration related α-syn or tau fibrils overexpressed in the membrane of a non-neuronal cell line (i.e. K562).

**Figure 6:**
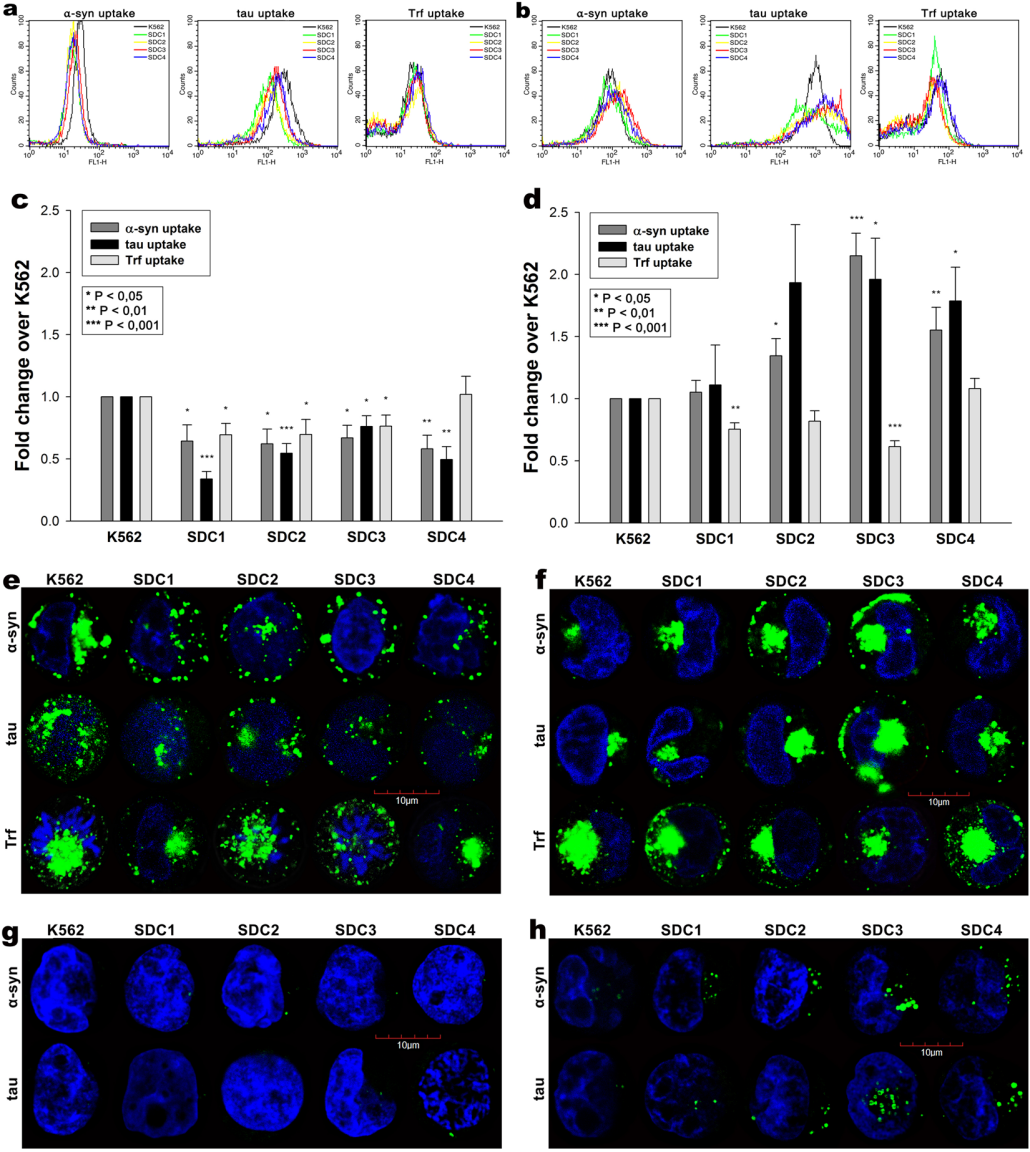
Cellular uptake of α−syn, tau into WT K562 cells and SDC transfectants following treatment with the monomers. WT K562 cells and SDC transfectants were treated with monomeric, FITC-labeled or unlabeled α−syn, tau at a concentration of 5 µM or Trf (25 µg/ml) for 1 or 18 h at 37 °C. Cellular uptake of α−syn, tau and Trf were then analyzed with flow cytometry and confocal microscopy. (**a**,**b**) Flow cytometry histograms showing intracellular fluorescence following 1 **(a)** or 18 h **(b)** incubation with either of the proteins (α−syn, tau or Trf). **(c,d)** Results of flow cytometric measurements. Detected fluorescence intensities are normalized to WT K562 cells treated with the respective fluorescent proteins (standards). The bars represent mean ± SEM of five independent experiments. Statistical significance vs protein-treated (either α−syn, tau or Trf) WT K562 cells (standards) was assessed by analysis of variance (ANOVA). **p < 0.01 vs standards; ***p < 0.001 vs standards. **(e,f)** CLSM visualization of SDC transfectants treated with fluorescently labeled proteins (α−syn, tau or Trf) for either 1 h (e) or 18 h (f) at 37 °C. Nuclei of cells were stained with DAPI. Representative images of three independent experiments are shown. Scale bar = 10 μm. **(g,h)** Internalization ThT-labeled, intracellular α−syn and tau fibrils in WT K562 cells and SDC transfectants, 1 (**g**) or 18 h (**h**) after treatment with the monomeric α−syn and tau. Representative images of three independent experiments are shown. Scale bar = 10 μm.

**Figure 7:**
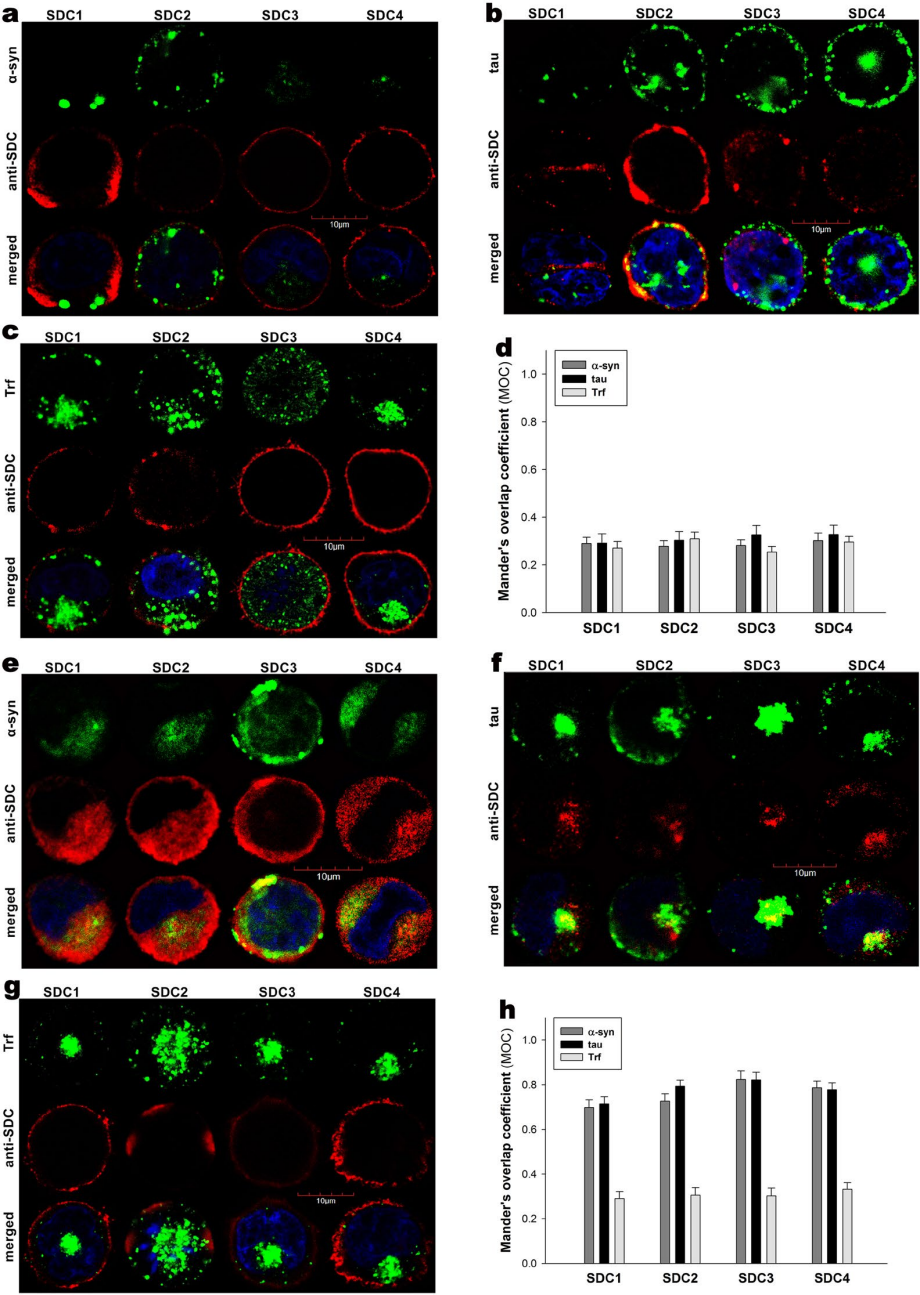
Colocalization studies after treatment with the monomers. SDC transfectants (established in K562 cells) were treated with either of the fluorescently labeled α−syn, tau monomers (at a concentration of 5 µM) or Trf (25 µg/ml) for 1 h or 18 h at 37 °C. After incubation, the cells were permeabilized and treated with the respective APC-labeled SDC antibody. Nuclei of cells were stained with DAPI and cellular uptake was then analyzed with CLSM. **(a**–**c)** CLSM images of SDC transfectants 1 h after treatment with either of the fluorescently labeled monomers (α−syn, tau) or Trf. Representative images of three independent experiments are shown. Scale bar = 10 μm. **(d**) Mander’s overlap coefficient (MOC) ± SEM for the overlap of SDCs with either of the fluorescently labeled monomers proteins (α−syn, tau) and Trf was calculated by analyzing 21 cellular images (7 images per sample, experiments performed in triplicate). **(e**–**g)** CLSM images of SDC transfectants 18 h after treatment with either of the fluorescently labeled monomers (α−syn, tau) or Trf. SDCs are labeled with the respective APC-labeled SDC antibody. Representative images of three independent experiments are shown. Scale bar = 10 μm. **(h)** MOC ± SEM for the overlap of SDCs with either of the fluorescently labeled monomers proteins (α−syn, tau) and Trf was calculated by analyzing 21 cellular images (7 images per sample, experiments performed in triplicate).

Uptake studies showed that while at 1 h of incubation the cellular uptake of monomeric α-syn and tau were lower in the SDC overexpressing lines, at 18 h, when fibril are already formed due to SDCs, SDC overexpression also triggered higher amount of α-syn or tau uptake (Fig. 6a–f). On the other hand, SDC transfectants – except for SDC4 – internalized less Trf at both time points, referring to affected clathrin-mediated endocytosis. Confocal microscopy also showed ThT-labeled aggregates intracellularly 18 h after treatment with monomeric α-syn or tau, especially in SDC transfectants, suggesting once SDCs trigger the formation of α-syn or tau fibrils, they also mediate the intracellular translocation of the fibrils (Fig. 6h). Further colocalization studies showed minimal overlap of SDCs with monomeric α-syn or tau at 1 h (Fig. 7a,b,d). However once in fibrillar state, α-syn or tau colocalize with SDCs, as shown by the increased MOCs for SDCs with α-syn or tau at 18 h of incubation (Fig. 7e,f,h). The strong colocalization of SDCs with α-syn or tau at 18 h of incubation suggests the common pathway SDCs and fibrillar α-syn or tau enter the cells. The overlap of SDCs with Trf remained quite minimal at both (i.e. 1 and 18 h) time points (Fig. 7c,d,g,h).

**Figure 8:**
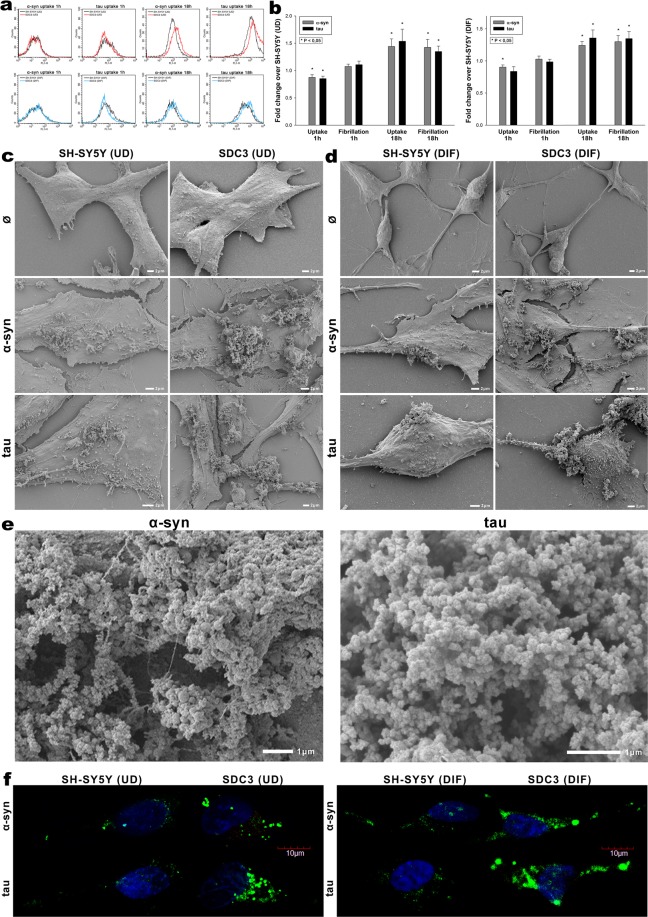
Effect of SDC3 overexpression on α−syn and tau fibrillation and uptake in SH-SY5Y cells. SDC3 overexpressing transfectants created in SH-SY5Y cells (either differentiated [DIF] or undifferentiated [UD]), along with WT SH-SY5Y cells (DIF or UD) were treated with fluorescently labeled (FITC) or unlabeled monomeric α−syn or tau at a concentration of 5 μM at 37 °C for 1 and 18 h. Cells were then processed for uptake and fibrillation studies. (**a**) Flow cytometry histograms representing α−syn and tau uptake of WT SH-SY5Y cells and SDC3 transfectants 1 and 18 h after treatment with the monomers. UD: undifferentiated; DIF: differentiated SH-SY5Y cells. **(b)** Fold change in α−syn and tau uptake and fibrillation due to SDC3 overexpression 1 and 18 h after treatment with the monomers. The bars represent mean ± SEM of five independent experiments. Statistical significance vs α−syn or tau treated WT SH-SY5Y cells (standards) was assessed by analysis of variance (ANOVA). *p < 0.05 vs α−syn or tau treated WT SH-SY5Y cells as standards. UD: undifferentiated; DIF: differentiated SH-SY5Y cells. **(c,d)** Scanning electron microscope visualization of cellular surface of α−syn and tau-treated WT SH-SY5Y cells, SDC3 transfectants. (**c**) Undifferentiated (UD) and (**d**) differentiated (DIF) SH-SY5Y cells. (**e**) Scanning electron microscope visualization of α−syn and tau fibrils formed on SDC3 transfectants. Representative images of three independent experiments are shown. Scale bar = 1 μm. **(f)** CLSM visualization of ThT labeled, intracellular α−syn and tau fibrils in WT SH-SY5Y cells and SDC3 transfectants 18 h after treatment with α−syn and tau monomers. Representative images of three independent experiments are shown. Scale bar = 10 μm. UD: undifferentiated; DIF: differentiated SH-SY5Y cells.

Overexpressing SDC3 in SH-SY5Y cells, either differentiated or undifferentiated, induced lower uptake of α-syn or tau monomers at 1 h (Fig. 8a,c), but significantly (p < 0.05) higher at 18 h of incubation, when fibrils are already formed, as revealed by both ThT fluorescence and electron microscopic studies (Fig. 8a–f). Thus 18 h after addition of α-syn or tau monomers, overexpressed SDC3 - either in undifferentiated or differentiated SH-SY5Y cells - triggered the fibrillation of both proteins. Electron microscopy also revealed widespread presence of mature fibrils on SDC3 overexpressing SH-SY5Y cells at 18 h (Fig. 8c–e), while confocal microscopic studies showed higher number of ThT-labeled aggregates in SDC3 transfectants at the same time point (Fig. 8f), suggesting that while inducing fibrillation, SDC3 also facilitate the uptake of fibrillar α-syn or tau.

## Discussion

The “protein-only hypothesis” proposed that small proteinaceous particles, termed prions, could be the infectious agents responsible for the transmission of spongiform encephalopathies, such as scrapie^88,89^. Despite initial skepticism, it is now widely accepted that prions are formed by a misfolded version of the cell-membrane prion protein that accumulates as aggregates^90,91^. Since other, more common neurodegenerative diseases, such as AD and PD, are similarly characterized by the accumulation of aggregated misfolded proteins, “prion-like” behavior of these pathologic aggregates has been hypothesized and investigated^92–94^. It is increasingly emerging that exogenous aggregates of α-syn or tau “infect” neighboring cells and propagate in experimental *in vitro* and *in vivo* settings^10^. Experimental studies have shown that the injection of tau and α-syn into animals induces neurons to form intracellular inclusions at the injection sites, from where they can also spread to distant brain regions^95–100^. These and other findings suggest that tau and other misfolded proteins have prion-like properties, and that spreading and seeding constitutes a central pathological mechanism for AD and other neurodegenerative diseases^94^. It is therefore important to understand the molecular mechanisms underlying the formation and cellular spreading of misfolded proteins^101^. Exploration of the cellular seeding and spreading phenomena of misfolded proteins in neurodegenerative diseases requires a strong understanding of protein uptake and endocytosis. In recent years, our research group has been exploring fundamental biological processes governing the SDC-mediated uptake of proteins^63,64^. First we described the SDC-mediated macropinocytic entry of peptidic drug delivery systems (DDSs) into the cells^36,63,68^. Utilizing our SDC overexpressing cellular models later we also revealed that SDCs, especially the neuron predominant SDC3, induce fibrillation and subsequent intracellular uptake of amyloid-β(1–42)^17^.

Considering the evidence backing up the HSPG-mediated entry of misfolded proteins, along with the increased expression of SDCs in human AD brains, we studied the uptake of α-syn and tau in our SDC cellular assays. K562 cells, a cell line expressing a minimal amount of membrane HSPGs and no caveolin-1, the major component of caveolae, are ideal cellular models to create stable SDC transfectants and assess the exact contribution of SDCs to the cellular uptake of α-syn and tau, without the interference of other HSPGs and caveolae-mediated endocytosis. As several observations suggest that the HS sulfation pattern is the same for every HS chain that a given cell synthesizes^27–30^, exploration of SDCs’ interactions with tau or α-syn in a given cell type (i.e. K562 cells) helped us to study whether the SDC type would also influence interactions with the misfolded proteins.

Cellular uptake assays in our SDC overexpressing cell lines showed that SDCs mediate the uptake of already fibrillar form of α-syn or tau, while α-syn or tau monomers enter the cells primarily through SDC independent routes. Besides their contribution to the uptake of fibrils, SDCs also induce the fibrillation of α-syn and tau. Once fibrils are formed, SDCs, especially the neuron predominant SDC3, facilitate the uptake of fibrillar α-syn and tau. It has become evident that the effect of SDC3 on fibrillation and cellular translocation α-syn and tau is independent of cell type and could be demonstrated in both neuronal and non-neuronal cell lines. Thus, the amount and type of SDC expressed in the membrane mattered more, then the cell type itself. Increased number of SDCs on the cell surface offer an ideal environment for the seeding and subsequent spreading of aggregation-prone proteins. Since HS fine structure appears to reflect the cellular source of the SDC and not the SDC type^31,102^, evidence gathered in multiple cell lines confirmed the importance of the SDC3 isoform type on interactions with tau or α-syn. Namely, the observed difference in SDCs’ interactions with α-syn and tau might go beyond the fine structure HS chains and should be also influenced by the other isoform specific regions of the SDC3 core protein.

The observation that overexpression of the neuron predominant SDC3 can indeed interfere with the uptake of α-syn and tau monomers, suggests that SDC3 overexpression can also support the propagation and cellular spread of fibrillar aggregates by affecting the clearance of monomeric α-syn and tau via classical endocytic routes. Attachment to SDCs (especially SDC3) therefore facilitates fibril formation as the local concentration increase of aggregation-prone α-syn and tau, the acidic molecular surrounding of HS chains and SDCs’ propensity to induce oligomerization of their ligands provide ideal conditions for aggregation^47,103–108^. The observed fibril triggering effect of SDCs, especially of SDC3, suggests that SDC binding could also enhance fibril maturation, thus creating a vicious circle of seeding and spreading. The neuronal predominance of SDC3 could also account for neurons being the main cellular targets of pathological protein fibrils.

Considering our data on the striking similarities on the interaction of neurodegeneration-related misfolded proteins (α-syn, tau and amyloid-β[1–42]) with SDCs, along with clinical findings on the increased expression of SDCs in human AD brains suggest that neurodegenerative disorders, especially AD, could be considered as “syndecanopathies”, namely the elevated level SDCs, especially the neuron predominant SDC3, creates favorable environment for fibrillation of aggregation-prone proteins (α-syn and tau or amyloid-β), besides facilitating their cellular translocation into neurons. As macromolecules or parasites entering the cells via SDCs can exert their biological activity intracellularly, cellular entry of pathological fibrillar assemblies via SDCs could be indeed a harmful uptake route that becomes dominant once fibrils are formed^64^.

In summary our paper reveals how SDCs, especially the neuron predominant SDC3, can mediate - regardless of cell type - the seeding and spreading of tau and α-syn. Our cellular data further supports recent clinical reports on the increased expression of SDCs in AD brains, providing fundamental biological evidence on the contribution of SDC overexpression to central molecular events in neurodegenerative disorders.

## Materials and Methods

### Fluorescent labeling aggregation of proteins

Recombinant tau-441 (2N4R isoform, purchased from rPeptide) was incubated for 5 days to form fibrils as described by Holmes *et al*.^13^. Briefly, 10 μM protein, in PBS 1 mM DTT pH 7.4, was mixed with low molecular weight heparin (0.05 mg/ml) and incubated with rotary agitation (400 rpm) at 37 °C for 5 days, while confirming fibrils formation was with Thiofavin T (ThT) assays and electron microscopy. Before use, fibrillization mixture was centrifuged and the resultant pellet resuspended in the PBS 1 mM DTT pH 7.4 without heparin to a stock solution. Formation of α-syn fibrils was induced as described by Ihse *et al*^87^. Briefly, α-syn (purchased from rPeptide) was dissolved in PBS to a concentration of 140 µM (~2 mg/ml), and incubated at 37 °C with rotary agitation (400 rpm) for 10 days, while monitoring fibrils formation with ThT assays and electron microscopy. After 10 days of incubation, the fibril solution was centrifuged at 20,000 × g for 30 min to separate the insoluble fibrils from smaller soluble aggregates and/or any monomers. The pellet was re-dissolved in PBS to a stock solution. Concentrations were determined by measuring absorbance using NanoDrop. Labeling of α-syn or tau fibrils was performed with the FITC or Alexa Fluor 633 labeling kits according to the manufacturer’s instructions (Thermo Fisher Scientific). The calculated yield of the labelling was 3.4 mol dye/tau, and 2.2 mol dye/α-syn. For all the experiments only freshly prepared sonicated fibrils (8 pulses of 30% amplitude) or freshly prepared monomers were used.

### SDC constructs, cell culture and transfection

SDC transfectants (in either K562 or SH-SY5Y cells) were created as described previously^17,36,68^.

### Differentiation protocol of SH-SY5Y cells

SH-SY5Y cells maintained at 37 °C in a humified 5% CO_2_ containing air environment were seeded at an initial density of 10^4^ cells/cm2 on 24 or 8 well plates in culture dishes (Corning) previously coated with 0.05 mg/ml collagen (Merck). All‐trans‐retinoic acid (RA, Sigma) was added the day after plating at a final concentration of 10 μM in Gibco™ Advanced MEM (Thermo Fischer Scientific) containing 2% FBS. The culture medium was changed every 2 days supplemented with fresh RA^109^. After 1 weeks in the presence of RA, cells were washed three times and incubated with 50 ng/ml BDNF (Sigma) in Advanced MEM (without serum) for 2 days, before treating the cells with α−syn or tau as described later. Neuronal differentiation was then justified by staining the cells with neuron specific human βIII-tubulin antibody (eBioscience™, cat. no. 14-4510-82) along with secondary Alexa Fluor 546-labeled goat anti-mouse IgG (H + L), cross-adsorbed secondary antibody (Invitrogen, cat. no. A-11003) and then visualizing the cells with confocal microscopy.

### Flow cytometry analysis of HS and CS expression

As HS was shown to attach α−syn and tau, HS expression of applied cell lines (WT K562 and SH-SY5Y cells, SDC transfectants) were measured with flow cytometry by using anti-human HS antibody (10E4 epitope, Amsbio; cat.no. 370255-1) and FITC- or Alexa Fluor 647 labeled goat anti-mouse IgG (Sigma); cat.no. SAB3701014, SAB4600333) according to the manufacturers’ protocols. SDC transfectants with almost equal amount of HS expression were selected for further uptake studies.

### Flow cytometry analysis of protein uptake

WT K562 and SH-SY5Y cells, SDC transfectants, along with SDC4 structural mutants were utilized to quantify internalization of the fluorescently (FITC or Alexa Fluor 633) labeled fibrils (α-syn or tau) or monomers, along with FITC-Trf. Briefly, 6 × 10^5^ cells/ml in DMEM/F12 medium (with 10% FCS) were incubated with the fluorescently labeled α-syn, tau or Trf (at a concentration of 5 μM monomer equivalent and 25 μg/ml, respectively), for various amounts of time (3 h in case of fibrils, while 1 and 18 h for the monomers) at 37 °C. After incubation the cells were washed twice in ice cold PBS and progressed towards flow cytometry. In the case of the FITC-labeled proteins, after incubation and washing, the cells (WT K562, SH-SY5Y and SDC transfectants) were resuspended in 0.5 ml of physiological saline. Equal volumes of this suspension and a stock solution of trypan blue (Merck KGaA; 500 μg/ml dissolved in ice-cold 0.1 M citrate buffer at pH 4.0) were allowed to mix for 1 min before the flow cytometric analyses. In this way, sample pH was lowered to pH 4.0, thereby optimizing the quenching effect of trypan blue to quench the extracellular fluorescence of surface bound fluorescent proteins^82^. In the case of the SDC4 mutants treated with Alexa Fluor 633-labeled proteins, extracellular fluorescence of surface attached α-syn or tau was removed by trypsinization according to the method described by Nakase *et al*.^110^. Cellular uptake was then measured by flow cytometry using a FACScan (Becton Dickinson). A minimum of 10,000 events per sample was analyzed. Viability of cells was determined by using propidium iodide in the cell suspension (10 µg/ml) and appropriate gating in a forward-scatter-against-side-scatter plot to exclude dead cells, debris, and aggregates.

### Microscopic visualization of uptake

Internalization of the fluorescently labeled (either FITC or Alexa Fluor 633) α-syn or tau fibrils or monomers, along with FITC-Trf, was visualized by confocal laser scanning microscopy (CLSM). WT SH-SY5Y and WT K562 cells, along with SDC transfectants and SDC4 mutants were grown on poly-D-lysine-coated glass-bottom 35-mm culture dishes (MatTek Corp.). After 24 h, the cells were preincubated in DMEM/F12 medium (supplemented with 10% FCS) at 37 °C for 30 min before incubation with the fluorescently labeled fibrils or monomers at a concentration of 5 μM monomer equivalent (or 25 μg/ml in case of Trf). After incubation, the cells were rinsed two times with ice-cold PBS, fixed in 4% paraformaldehyde (Sigma) and nuclei were stained with DAPI (1:5000, Sigma) for 5 min. For colocalization studies, after fixation, the cell membranes were permeabilized (1% Triton X-100), followed by 1 h of treatment with APC-labeled SDC antibodies (1:100) with or without either of the Alexa Fluor 546-labeled flotillin antibodies (flotillin-1 or 2, all Santa Cruz Biotech, cat.no. sc-74566 AF546; sc-28320 AF546). The samples were then rinsed three times with PBS containing 1% goat serum and 0.1% Triton X-100, then stained with DAPI (1:5000) for 5 min, washed three times with PBS and embedded in Fluoromount G (SouthernBiotech). Distribution of fluorescence was then analyzed on an Olympus FV1000 confocal laser scanning microscope equipped with three lasers. A laser diode (excitation, 405 nm) and a band-pass filter (420–480 nm) were used to capture the signal recorded as blue; an argon laser (excitation, 488 nm) and a bandpass filter (505–530 nm) were used to capture the signal recorded as green; and finally, a helium/neon laser (excitation, 543 nm) and a band-pass filter (550–625 nm) were used to capture the signal recorded as red. Sections presented were taken approximately at the mid-height level of the cells. Photomultiplier gain and laser power were identical within each experiment. The Olympus Fluoview software (version 4.2b) was used for image acquisition and analysis. For visualizing internalization of ThT-labeled fibrils, WT K562 cells and SDC transfectants grown on poly-D-lysine-coated glass-bottom 35-mm culture dishes were incubated with α-syn, tau at a concentration of 5 µM monomer equivalent (in DMEM/F-12 without Phenol Red) at 37 °C for 18 h, then treated with Thioflavin T (ThT, Sigma) at a concentration of 25 µM for 10 min at 37 °C and rinsed two times with ice-cold PBS. After fixation in 4% paraformaldehyde (Sigma), nuclei were stained with DAPI (1:5000) for 5 min, the after three washing with PBS, the samples were embedded in Fluoromount G and distribution of fluorescence was analyzed on an Olympus FV1000 confocal laser scanning as described above. For colocalization analyses (SDCs with α-syn, tau or Trf; SDCs with flotillins, SDC3 with α-syn, tau or flotillins), the Mander’s overlap coefficient (MOC) was calculated by analyzing 21 cellular images (7 images per sample, experiments performed in triplicate) with the built-in colocalization module of Olympus Fluoview software (version 4.2b).

### Co-immunoprecipitation experiments

Stable SDC3 transfectants or WT SH-SY5Y cells were incubated with or without FITC-labeled α−syn or tau fibrils at a concentration of 5 µM monomer equivalent for 3 h at 37 °C. After incubation the cells were washed twice with ice cold PBS and treated with cold Pierce IP lysis buffer. Then the cells were scrapped off to clean Eppendorf tubes, put on a low-speed rotating shaker for 15 min and centrifuged at 14,000 g for 15 min at 4 °C. The supernatants were then transferred to new tubes and combined with 5 µg of the human SDC3 or SDC4 antibody (R&D Systems; cat.no. AF3539 and AF2918F) or 5 μg of flotillin-1 and 2 (FLOT1 and 2) antibody (Santa Cruz Biotechnology; cat.no. sc-28320, sc-74566). The antigen sample/SDC3, SDC4 antibody or antigen sample/FLOT1, FLOT2 mixture was then incubated for overnight at 4 °C with mixing. The antigen sample/SDC3, SDC4 antibody and antigen sample/FLOT1 or 2 antibody mixture then were added to a 1,5 ml microcentrifuge tube containing pre-washed Pierce Protein A/G Magnetic Beads (Thermo Fisher Scientific, cat.no. 88802) and after incubation at room temperature for 1 hour with mixing, the beads were then collected with a MagJET Separation Rack magnetic stand (Thermo Fisher Scientific) and supernatants were discarded. To elute the antigen, 100 µl of SDS-PAGE reducing sample buffer was then added to the tubes and samples were heated at 96 °C for 10 minutes in 1% SDS and the samples were proceeded to SDS-PAGE.

### Thioflavin T binding assays

WT K562, SH-SY5Y cells and SDC transfectants seeded into black-sided, clear bottom 96-well microplates (Corning) at a density of 1,5 × 10^5^ cells/well in 100 µl of DMEM/F-12 (without Phenol Red) were exposed to monomeric α−syn and tau at a concentration of 5 µM for various amounts of time (1, 3, 6 and 18 h) at 37 °C. After the incubation periods, Thioflavin T (ThT) was added to the α−syn or tau-treated cells at a concentration of 15 µM and after 10 min of incubation fluorescence was measured with Cytation™ 3 Multi-Mode reader (BioTek Instruments) using an excitation wavelength of 440 nm and an emission of 480 nm. Photomultiplier gain was set at 50. Fluorescence measurements are made from the bottom of the plate, with the top being sealed with an adhesive plate sealer to prevent evaporation. The fold change in ThT fluorescence intensity over background ThT signal was calculated by dividing the fluorescence intensity of the α−syn and tau-treated cells incubated with ThT by the respective fluorescence intensity of the ThT-incubated (same) cell line untreated with α−syn and tau.

### Scanning electron microscopy of surface attachment and fibrillation

WT SH-SY5Y and WT K562 cells, along with SDC transfectants were grown on poly-D-lysine-coated glass-bottom 35-mm culture dishes. After 24 h, the cells were preincubated in DMEM/F12 medium (supplemented with 10% FCS) at 37 °C for 30 min before incubation with either α−syn or tau fibrils or monomers (at a concentration of 5 μM monomer equivalent) for various amounts of time (10 min and 3 h for the fibrils and 1, 6 and 18 h for the monomers). The cells were then rinsed two times with ice-cold PBS, then fixed in 2.5% glutaraldehyde and 0.15% alcian blue 8GX (Sigma) for 1 hour. After post-fixation in 1% OsO_4_ (Sigma) for 1 hour, the samples were dehydrated in aqueous solutions of increasing ethanol concentrations, critical point dried, covered with 10 nm gold by a Quorum Q150T ES sputter and observed in a JEOL JSM-7100F/LV scanning electron microscope.

### Promoting undersulfation

To study the effect of proteoglycan sulfation on fibril uptake, cells were incubated with 60 mM sodium chlorate (NaClO_3_; Sigma) for 48 h, the processed for the flow cytometry and microscopy studies as described above.

### Statistical analysis

Results are expressed as means ± standard error of the mean (SEM). Differences between experimental groups were evaluated by using one-way analysis of variance (ANOVA). Values of p < 0.05 were accepted as significant.

## Supplementary information

Supplementary Info
