# Supplementary material for: Contribution of syndecans to cellular uptake and fibrillation of α-synuclein and tau
Source: Sci Rep. 2019 Nov 12;9:16543. doi: 10.1038/s41598-019-53038-z (PMC6851098; doi:10.1038/s41598-019-53038-z)
Supplement: Supplementary file 1 — Supplementary Info [file 41598_2019_53038_MOESM1_ESM.docx]

Supplementary Information

**Contribution of syndecans to cellular uptake and fibrillation of α-synuclein and tau**

**Anett Hudák^1^, Erzsébet Kusz^1^, Ildikó Domonkos^2^, Katalin Jósvay^2^, Alpha Tom Kodamullil^3^, László Szilák^4^, Martin Hofmann-Apitius^3^, and Tamás Letoha^1^***

^1^Pharmacoidea Ltd., Szeged, H-6726, Hungary

^2^Biological Research Centre of the Hungarian Academy of Sciences, Szeged, H-6726, Hungary

^3^Fraunhofer Institute for Algorithms and Scientific Computing (SCAI), Sankt Augustin, 53754, Germany

^4^Szilak Laboratories, Bioinformatics and Molecule-Design, Szeged, H-6723, Hungary

*corresponding author; e-mail: [tamas.letoha@pharmacoidea.eu](mailto:*tamas.letoha@pharmacoidea.eu), phone: +36 30 2577393


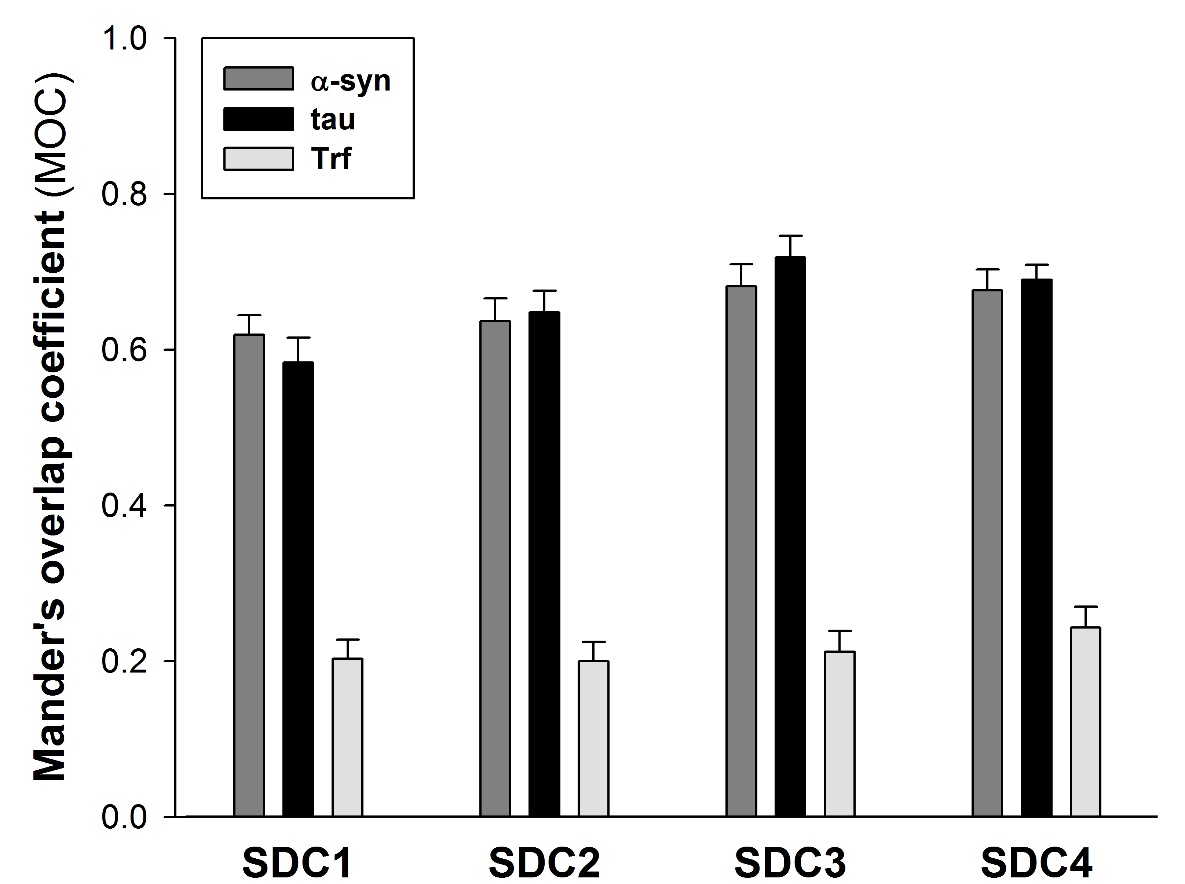


**Supplementary Fig. S1.** The Mander’s overlap coefficients (MOCs) calculated in colocalization studies in fibril-treated (α-syn or tau) stable SDC transfectants (created in K562 cells), as described in Materials and Methods. The MOC values for the overlap of SDCs with either of the fibrils (α-syn or tau) were calculated by analyzing 21 images (7 images per sample, experiments performed in triplicate) with the Olympus Fluoview software (version 4.2b). The bars represent the mean of MOC ± SEM for the overlap of SDCs with either of the α-syn, tau fibrils.

**
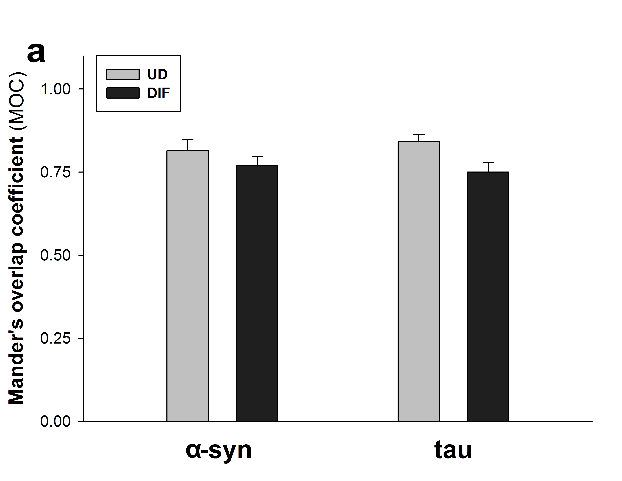
**

**
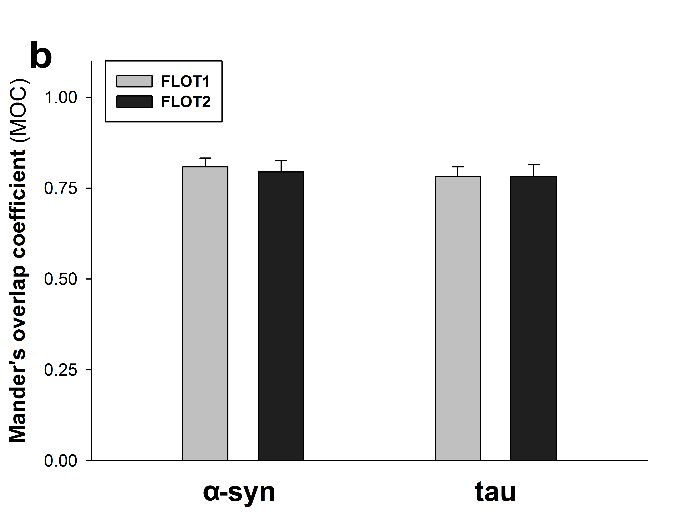

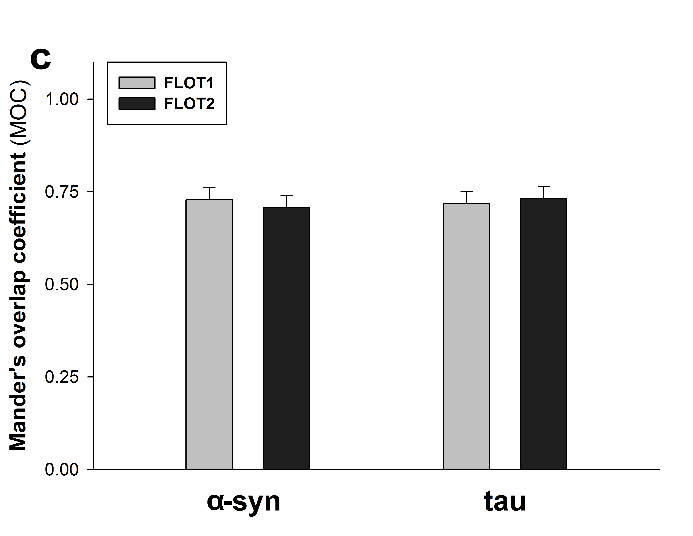
**

**Supplementary Fig. S2.** The Mander’s overlap coefficients (MOCs) calculated in colocalization studies in SH-SY5Y cells. SH-SY5Y cells – differentiated (DIF) or undifferentiated (UD) – was treated with the FITC-labeled fibrils, along with SDC3 or FLOT antibodies as described in the Materials and Methods. The MOCs were calculated by analyzing 21 cellular images (7 images per sample, experiments performed in triplicate) with the Olympus Fluoview software (version 4.2b). (**a**) MOC ± SEM for the overlap of SDC3 with either of the α-syn, tau fibrils. (**b,c**) MOC ± SEM for the overlap of FLOT1 or 2 with either of the α-syn, tau fibrils obtained on undifferentiated (**b**) or differentiated (**c**) SH-SY5Y cells.
